# Interspecific Association and Environmental Interpretation of Dominant Species in Shrub Layer of *Pinus massoniana* Community on Chinese Islands

**DOI:** 10.1002/ece3.70647

**Published:** 2024-12-05

**Authors:** Jihong Xiao, Qingyan Wen, Zhifei Zhong, Xiting Lin, Yingxue Wang, Yanqiu Xie, Feifan Weng, Qingya Deng, Guochang Ding, Chuanyuan Deng

**Affiliations:** ^1^ College of Landscape Architecture and Art Fujian Agriculture and Forestry University Fuzhou China

**Keywords:** interspecific association, island, niche, *Pinus massoniana* communities, shrub layer

## Abstract

Understanding the factors driving species coexistence and competition in the shrub layer of semi‐natural forests is crucial for effective forest management and conservation. However, there is limited knowledge about the interspecific associations of the main species in the shrub layer of *Pinus massoniana* communities in the semi‐natural forest of Sandu Gulf, Ningde, Fujian Province, China. Therefore, this study aimed to investigate the influence of the abiotic environment on plant communities within the semi‐natural forest of *P. massoniana* on the islands of Sandu Gulf. By exploring these interspecific associations, we sought to provide a more accurate understanding of the causes and processes of species coexistence and competition. The ultimate goal of this project was to offer a reference basis for optimizing the shrub layer structure in *P. massoniana* (plantation) forests. We found that (1) *Heptapleurum heptaphyllum* was the most dominant species in the shrub layer, while *Smilax china* demonstrated the broadest range of environmental adaptability and correspondingly broader niche than other species. (2) Our analysis revealed a predominance of positive associations among the dominant species in the shrub layer. However, the overall association was not significant, with relatively small positive and negative associations between species pairs. The significant test rate was low, and the *NRI* exhibited a non‐significant aggregation. These findings suggest that the plant community in the shrub layer has not yet reached its most stable stage. (3) We also observed that the distribution of dominant species in the shrub layer was primarily affected by factors such as total potassium, pH, available potassium, and light (canopy density). (4) Soil pH value decreased gradually as sampling points moved inward away from the coastline, and island isolation, temperature, and precipitation indirectly affected the species' importance in the shrub layer. Considering the intense competition among the understory species, it is crucial for conservation efforts to prioritize species pairs with reduced ecological niche overlap or significant positive associations. This approach will effectively reduce competition and contribute to the maintenance of stability in the shrub layer. This study provides a theoretical basis for environmental and vegetation restoration, optimizing the species composition of island plantation forests, rationalizing plant composition, and implementing effective operation and management practices for local *P. massoniana* forests.

## Graphical abstract

*Smilax china* had the largest niche breadth and the strongest environmental adaptability, and the local community is still in the development stage and is not stable enough. The distribution of dominant species in the shrub layer was primarily affected by total potassium, pH, and available potassium. There was a significant quadratic relationship between total and available potassium. Furthermore, the distribution of both Heptapleurum heptaphyllum and *Melastoma malabathricum* was strongly affected by light (canopy density). Soil pH value was found to decrease gradually as sampling points moved inward away from the coastline, the increase in island isolation and slopes gradually became ubac, rather than adret. Slope direction, distance from the coastline, island isolation, temperature and precipitation indirectly affected the important value of species in shrub layer by directly affecting the pH. Island isolation, temperature, and precipitation also indirectly affect the important value of species in shrub layer by directly influencing soil chemical content, such as soil organic matter (SOM), nitrogen (N), phosphorus (P), and potassium (K).

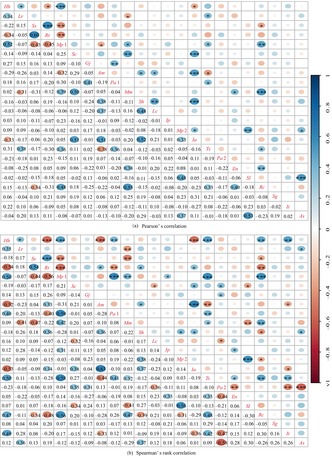

## 1 Introduction

The interspecific association of plant communities is an important quantitative and structural characteristic of plant communities (Álvarez‐Yépiz, Búrquez, and Dovciak 2014) and has attracted considerable attention in recent years as a hotspot of ecological research. Studying the interspecific associations of communities can provide an understanding of community succession and interspecific relationships (Zhang et al. 2015), and is also important for exploring tree species pairing and achieving scientific and rational forest management (Jiang, Lu, and Pang 2014). Species distribution is often influenced by differences in community habitat, creating interspecific association (Peng et al. 1999). There are significant differences in the habitats of inner and outer islands. As top zonal communities, the conclusions of studies on the *Acacia confusa* communities differ greatly on inner and outer islands. For example, the dominant species in the shrub layer of *A. confusa* community on Pingtan Island (Ma et al. 2022), an outer island, was young trees of *A. confusa*. On the other hand, on Langqi Island (Xiao et al. 2022), an inland island with little difference in latitude and longitude from Pingtan Island, the dominant species in the shrub layer of *A. confusa* community was *Sageretia thea* , and the proportion of young trees of *A. confusa* in the shrub layer was extremely low. This indicates that the dominant species of the same community can vary greatly in different habitats, suggesting that there are differences in interspecific associations within the shrub layer. However, current studies of interspecific associations have less frequently included environmental factors in their results and analyses. Therefore, how to accurately explain the causes of species coexistence and competition has become the subject of current research. Topography and soil are the main factors to explain the distribution characteristics of community species (Wang, Bi, and Pang 2018; Elias et al. 2019). In island ecosystems, the island isolation is also an important influencing factor (Helmus, Mahler, and Losos 2014). Chen et al. (2021) and Jiang and Zhang (2021) explained the leading factors affecting the distribution and interspecific association of the dominant species in the study area through canonical correspondence analysis (CCA) and redundancy analysis (RDA) respectively, making the conclusions more intuitive and scientific. Therefore, studying the relationship between species distribution and the environment can not only correctly grasp the dominant environmental factors affecting the community structure and distribution, but also provides a more scientific explanation of the causes affecting interspecific associations in the community, ultimately assisting in the classification of ecological species groups. However, these studies also only used the results to predict influencing factors and did not validate them by collecting and analyzing specific environmental factors. Therefore, this study will collect specific environmental factors and analyze them using a combination of CCA or RDA to provide a more accurate explanation.

Island ecosystems are frequently threatened by typhoons and waves, with poor soil fertility. Due to their isolation, island ecosystems are vulnerable to damage and have difficulty recovering from extreme disturbance (Chi et al. 2015). To mitigate this risk and reduce the ecological vulnerability of islands, scientists and governments often plant plantations designed to foster soil development and reduce erosion (Chi et al. 2015, 2017; Wang, Bi, and Pang 2018). However, plantations have many disadvantages, such as simple community structure and characteristically lacking understories. In contrast, natural or semi‐natural forests are richer and more diverse in understories. With the evolutionary renewal of the community, the dominant species suitable for survival in the community gradually manifest themselves and together constitute a relatively stable community structure. Therefore, studying the interspecific association of main species in the shrub layer of semi‐natural forests can help understand their species coexistence mechanisms, community structure and stability characteristics, and can also provide reference for optimizing the shrub layer structure of unstable *P. massoniana* plantations and the operation and management of local *P. massoniana* forests.

The islands of the Sandu Gulf, located in the inland sea, are situated between the South Subtropical Zone and the Middle Subtropical Zone, with most of its area covered by wild *P. massoniana* forests, which are highly adaptable and drought‐resistant (Cui et al. 2014; Xiao, Lai, et al. 2023). *P. massoniana* covers more area than any other tree species and is representative of most forest in the region. The species is of irreplaceable ecological value. The *P. massoniana* forests on islands of the Sandu Gulf are semi‐natural communities largely formed artificially either by aerial seeding or artificial afforestation over the last century, which are subject to the constraints of local policies, with relatively small human influence (Xiao, Lai, et al. 2023). At present, the *P. massoniana* forests are in the stage of succession to evergreen mixed forest or evergreen broad‐leaved forest (Xiao, Lai, et al. 2023). Due to the allelopathy caused by *Dicranopteris pedata*, there are very few species in the herbaceous layer, but the understory shrub layer is the most species‐rich layer in the local *P. massoniana* community, maintaining the balance and stability of the ecosystem, and is ecologically significant (Xiao, Lai, et al. 2023). In addition, the shrub layer also links the herbaceous and the tree layer, with substitution and connection functions (Liu et al. 2020). However, there is a lack of research on the interspecific associations of plants in the shrub layer of the semi‐natural forest in *P. massoniana* on Sandu Gulf, and we have not figured out how these semi‐natural communities may form or the environmental parameters that may influence their structure.

Therefore, it is necessary to study the shrub layer of *P. massoniana* semi‐natural forest on Sandu Gulf islands, especially the interspecific associations and environmental interpretations of dominant species in the shrub layer. Motivated by this, we explored the shrub layer of these semi‐natural communities by surveying forests across six islands within the Sandu Gulf. We aim to address the following scientific questions: (1) What are the characteristics of the ecological niche of the main species in the shrub layer of the semi‐natural forest in *P. massoniana* on Sandu Gulf? (2) What are the characteristics of the interspecific associations of the main species in the shrub layer of the semi‐natural forest in *P. massoniana* on Sandu Gulf? (3) What are the main abiotic factors affecting the distribution and interspecific association characteristics of the main species in the shrub layer of the semi‐natural forest in *P. massoniana* on Sandu Gulf? By reasonably addressing these scientific questions, this study aims to reveal the niche characteristics, interspecific association characteristics, community stability, and major non‐biological factors influencing species distribution in the shrub layer of the *P. massoniana* semi‐natural forests in the region, providing a theoretical basis for environmental and vegetation restoration, optimizing the species composition of island plantations, and the rational construction and operation of local *P. massoniana* forests.

## 2 Materials and Methods

### 2.1 Research Area Overview

Sandu Gulf is located to the southeast of Ningde City within Fujian Province. It is the midpoint of China's “Golden Coastline” (18,400 km), approximately 30 km away from Ningde. It is a world‐class natural fine deep‐water port with 126 islands, including 17 residential ones, among which Sandu Island is the largest, covers about 27.74 km^2^. Sandu Island is the seat of the Sandu Town government. The study area has a typical subtropical marine monsoon climate and is hilly and predominated by mainly red and yellow soils (Xiao, Lai, et al. 2023). The secondary *P. massoniana* coniferous forest and *P. massoniana* coniferous and broad‐leaved mixed forest are the most widely distributed forests on the island. The main group species are *P. massoniana* , *Heptapleurum heptaphyllum*, and *Adinandra millettii*. The main auxiliary species are *Symplocos sumuntia*, *Toxicodendron succedaneum* , and *Litsea rotundifolia* var. *oblongifolia*. Except for young trees, there are mainly *Rhodomyrtus tomentosa* , *Mussaenda pubescens*, *Gardenia jasminoides* , and other dominant shrub layer species (Xiao, Lai, et al. 2023; Xiao, Zhong, et al. 2023).

### 2.2 Community Study Survey

**FIGURE 1:**
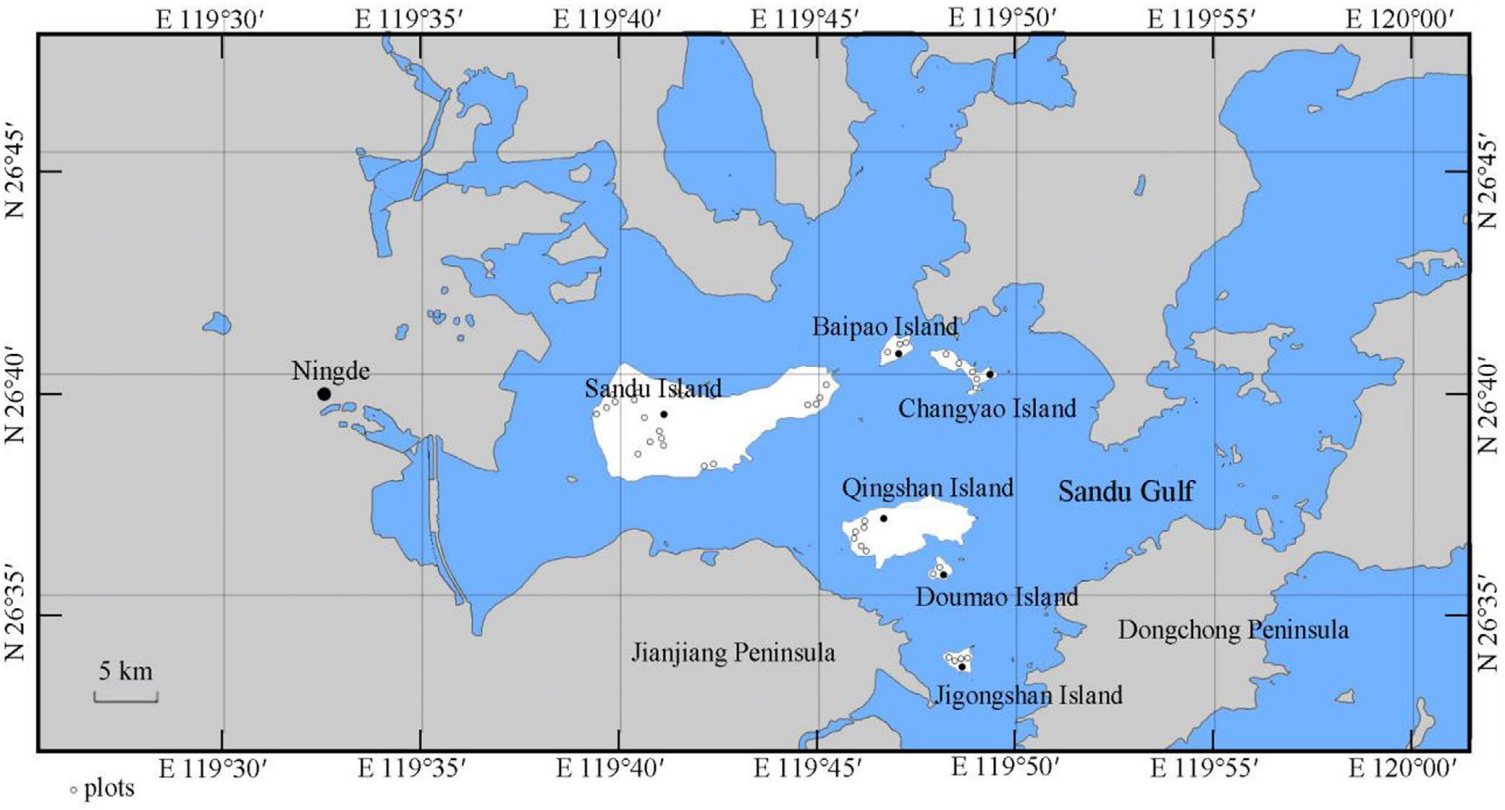
Locations of six islands in the Sandu Gulf.

Based on a field survey conducted from June to August 2022, the Relevé method (Song 2017) was used to set the sampling plots across six inhabited islands with large areas and high forest coverage, namely Sandu Island, Qingshan Island, Changyao Island, Jigongshan Island, Baipao Island, and Doumao Island. A total of 40 20 m × 20 m plots were established (set 20, 6, 5, 4, 3, and 2 plots in 6 islands, respectively, Figure 1) within *P. massoniana* communities. One shrub quadrat with an area of 5 m × 5 m was set up in each of the four corners of each forest sampling plot, meaning we sampled a total of 160 shrub quadrats. Species identity (based on *Flora of China* (http://www.iplant.cn/foc, accessed on December 1, 2022)), plant height, coverage, quantity, and other information on each individual shrub including bamboo, woody vines, and young trees with a height of < 3 m and a chest diameter of < 3 cm were recorded. A GPS and compass were used to record longitude and latitude, elevation, slope aspect, slope gradient, and slope position for the sampling plot. Satellite imagery and longitude and latitude measurements are used to calculate the distance from the sample plot to the coastline, the distance to the nearest island, and the distance to the mainland. These last two terms were used to generate an “island isolation factor” for each island. Slope was divided into upper, middle, and lower slopes. Slope aspect was considered to fall into one of four categories: adret (157.5°~247.5°), semi‐adret (112.5°~157.5°, 247.5°~292.5°), ubac (0°~67.5°, 337.5°~360°), and semi‐ubac (67.5°~112.5°, 292.5°~337.5°) (Li et al. 2018). The crown density was estimated in Photoshop 2022, following digital image on canopy closure determination method (CCPS) laid out in Qi, Luo, and Zhao (2009). We obtained the average annual temperature, precipitation, and wind speed of each sample plot from 2018 to 2020. The temperatures (1‐km monthly mean temperature dataset for China (1901–2021)) and precipitation (1‐km monthly precipitation dataset for China (1901–2021)) were obtained from the National Qinghai‐Tibet Plateau Scientific Data Center (https://data.tpdc.ac.cn, accessed on December 24, 2022). Wind speed (1‐km monthly mean wind speed dataset for China (2001–2020)) were obtained from the National Earth System Science Data Center, National Science & Technology Infrastructure of China (http://www.geodata.cn, accessed on December 24, 2022). The basic information of sample plots is shown in Table 1. Soil was collected using a five‐point sampling method at a depth of 25 cm from each plot, ground, sieved, and measured three times to determine nine indicators: pH value, total potassium, available potassium, total phosphorus, available phosphorus, total nitrogen, alkali‐hydrolyzed nitrogen, soil organic matter, and total amount water‐soluble salt (Lu 2000). The soil information is shown in Table 2.

**TABLE 1:** General information of sampling plot.

Sample plot no.	Longitude	Latitude	Elevation/(m)	Slope aspect	Slope/(°)	Topographic position	Crown density	Distance from coastline/(km)	Distance to the nearest island/(km)	Distance to the mainland/(km)	Air Temperature/(°C)	Precipitation/(mm/a)	Wind speed/(m/s)
S1	119°40′47.66″	26°39′07.25″	42.4	SE160 (A)	30	Middle	0.75	1.438	2.844	3.699	20.206	1319.333	1.072
S2	119°40′47.12″	26°39′09.55″	81.1	SE160 (A)	30	Up	0.75	1.508	2.824	3.761	19.864	1328.361	1.072
S3	119°39′33.26″	26°39′53.18″	178.4	N353 (U)	30	Up	0.80	0.596	1.455	3.488	19.989	1327.944	0.964
S4	119°39′20.11″	26°39′51.20″	71.9	NW312 (SU)	30	Middle	0.75	0.302	1.238	3.222	19.989	1327.944	0.952
S5	119°39′32.59″	26°39′57.99″	123.2	W261 (SA)	37	Up	0.68	0.515	1.573	3.395	19.989	1327.944	0.964
S6	119°40′22.00″	26°39′43.00″	175.5	SE155 (SA)	32	Middle	0.80	1.652	2.316	4.659	19.481	1330.194	1.097
S7	119°40′16.47″	26°40′10.93″	40.7	NW289 (SU)	24	Low	0.75	0.911	2.657	4.210	20.042	1331.361	1.063
S8	119°40′10.60″	26°40′07.14″	96.0	NE23 (U)	23	Low	0.80	1.096	2.458	4.126	19.481	1330.194	1.052
S9	119°44′53.67″	26°40′25.00″	126.0	SW205 (A)	30	Up	0.55	0.512	1.738	4.035	19.436	1320.167	0.767
S10	119°44′35.47″	26°39′58.77″	157.2	S180 (A)	30	Up	0.93	0.491	2.024	4.549	19.894	1325.778	0.690
S11	119°44′46.93″	26°40′00.68″	133.0	SW191 (A)	30	Middle	0.95	0.368	1.749	4.615	19.892	1322.500	0.767
S12	119°44′44.44″	26°39′59.58″	140.9	NE47 (U)	30	Middle	0.90	0.380	1.800	4.622	19.892	1322.500	0.690
S13	119°42′04.34″	26°38′33.02″	109.1	SW224 (A)	28	Low	0.60	0.152	3.428	3.287	19.991	1323.150	1.074
S14	119°41′55.43″	26°38′29.77″	109.1	SW197 (A)	35	Low	0.60	0.031	3.350	3.251	20.043	1323.280	1.094
S15	119°40′46.81″	26°39′11.85″	92.1	SE119 (SA)	26	Middle	0.80	1.581	2.815	3.829	19.864	1328.361	1.072
S16	119°40′40.70″	26°39′06.44″	49.4	SE151 (SA)	32	Low	0.65	1.420	2.650	3.626	20.206	1319.333	1.072
S17	119°40′19.56″	26°38′53.23″	55.0	SE115 (SA)	22	Low	0.60	1.057	2.136	3.120	20.050	1326.528	0.929
S18	119°39′09.64″	26°39′46.10″	36.2	NW294 (SU)	22	Low	0.58	0.181	1.016	3.102	19.989	1327.944	0.946
S19	119°41′11.56″	26°40′14.92″	89.3	NE39 (U)	27	Low	0.88	0.410	1.185	4.185	19.953	1332.000	1.111
S20	119°41′16.77″	26°40′12.60″	64.0	NE35 (U)	30	Low	0.85	0.446	1.072	4.217	19.953	1332.000	1.123
Q1	119°45′49.49″	26°36′45.54″	83.2	SW197 (A)	28	Middle	0.70	0.373	0.694	2.218	19.978	1304.139	1.247
Q2	119°45′54.47″	26°36′44.06″	74.4	SW212 (A)	26	Middle	0.80	0.376	0.825	1.975	19.978	1304.139	1.246
Q3	119°45′49.49″	26°37′14.24″	160.7	NW342 (U)	24	Middle	0.67	0.364	1.197	2.823	19.831	1305.028	1.214
Q4	119°45′51.85″	26°37′19.87″	92.4	N7 (U)	34	Middle	0.80	0.205	1.375	2.991	19.831	1305.028	1.215
Q5	119°45′32.05″	26°37′02.60″	36.9	SW219 (A)	34	Low	0.80	0.118	0.631	2.231	19.901	1304.810	1.220
Q6	119°45′33.29″	26°37′04.47″	58.1	SW242 (A)	35	Middle	0.80	0.185	0.698	2.301	19.893	1304.760	1.224
C1	119°48′34.85″	26°40′11.21″	26.0	SW208 (A)	38	Up	0.65	0.054	1.515	2.206	19.908	1299.540	0.857
C2	119°48′43.84″	26°40′35.66″	49.2	S175 (A)	36	Middle	0.95	0.250	0.761	1.570	19.883	1301.361	0.795
C3	119°48′02.29″	26°41′07.33″	54.2	N351 (U)	26	Low	0.93	0.175	1.417	0.546	19.682	1311.060	0.683
C4	119°48′20.11″	26°40′57.27″	56.6	NE71 (SU)	33	Low	0.65	0.079	1.143	0.734	19.772	1308.833	0.715
C5	119°48′43.16″	26°40′48.40″	16.2	NE55 (U)	27	Low	0.55	0.029	0.541	1.220	19.892	1303.472	0.795
J1	119°48′24.42″	26°34′14.67″	104.7	E88 (SU)	37	Up	0.70	0.321	0.961	1.816	19.681	1270.472	1.838
J2	119°48′35.68″	26°34′15.09″	44.6	E83 (SU)	24	Middle	0.80	0.140	0.891	2.066	19.681	1270.472	1.819
J3	119°48′08.40″	26°34′16.59″	47.7	NE38 (U)	38	Middle	0.70	0.228	1.239	1.561	19.688	1273.160	1.842
J4	119°47′59.29″	26°34′17.35″	19.3	N3 (U)	28	Low	0.78	0.140	1.430	1.440	19.695	1274.560	1.854
B1	119°46′31.03″	26°41′06.06″	99.5	SW225 (A)	34	Up	0.68	0.328	1.737	2.078	19.614	1321.190	0.703
B2	119°47′01.63″	26°41′23.15″	45.8	SE127 (SA)	22	Up	0.75	0.125	0.988	1.099	19.617	1321.500	0.650
B3	119°47′03.07″	26°41′23.62″	44.6	E83 (SU)	24	Middle	0.80	0.084	0.957	1.062	19.681	1270.472	1.819
D1	119°47′39.27″	26°36′09.74″	57.0	NW282 (SA)	31	Up	0.85	0.227	1.073	2.134	19.928	1283.833	1.608
D2	119°47′45.28″	26°36′15.55″	63.0	NW291 (SA)	23	Low	0.75	0.130	0.983	2.375	19.928	1283.833	1.617

Abbreviations: A, Adret; B, Baipao Island; C, Changyao Island; D, Doumao Island; J, Jigongshan Island; Q, Qingshan Island; S, Sandu Island; SA, Semi‐adret; SU, Semi‐ubac; U, Ubac. The same below.

**TABLE 2:** Chemical indicators of soil.

Sample plot no.	pH	K g/kg	K mg/kg	T‐P g/kg	P mg/kg	T‐N g/kg	N mg/kg	SOM g/kg	S g/kg
S1	4.55	1.17	73.71	0.09	2.31	1.03	172.12	31.48	1.36
S2	4.66	0.41	52.89	0.06	1.40	0.72	157.09	23.36	1.62
S3	4.65	4.71	76.73	0.19	1.33	1.47	119.25	38.39	0.97
S4	4.66	3.19	44.26	0.34	3.95	1.67	161.40	43.98	0.97
S5	4.64	3.19	51.64	0.10	1.55	0.74	141.96	20.99	1.51
S6	4.44	6.95	32.71	0.26	1.10	1.38	179.98	47.05	1.34
S7	4.41	4.78	91.24	0.21	1.43	1.84	224.79	48.87	1.78
S8	4.30	5.04	45.62	0.11	1.80	1.33	214.71	45.98	1.46
S9	4.36	9.84	45.62	0.10	0.96	1.24	163.96	48.45	1.81
S10	4.60	3.68	51.89	0.12	1.51	1.22	152.48	37.95	0.87
S11	4.61	3.16	74.07	0.16	0.92	1.31	167.87	41.78	0.49
S12	4.61	3.32	102.86	0.15	1.24	1.64	231.32	42.81	1.49
S13	4.62	7.14	84.01	0.16	1.35	0.96	140.50	39.87	1.02
S14	4.67	8.82	84.01	0.19	1.68	1.38	180.03	41.16	1.64
S15	4.49	3.69	112.70	0.17	1.12	1.65	148.91	41.20	1.37
S16	4.67	2.07	55.02	0.09	1.31	1.06	136.17	29.22	1.69
S17	4.45	2.05	74.81	0.11	3.17	0.87	178.88	22.06	0.82
S18	4.68	7.59	23.69	0.23	0.85	1.36	166.64	45.70	1.07
S19	4.44	6.70	44.71	0.19	1.02	1.79	192.54	52.85	1.22
S20	4.22	9.06	44.48	0.21	1.30	1.30	204.37	34.74	0.50
Q1	4.92	2.66	55.58	0.15	1.63	1.32	80.06	37.04	1.36
Q2	5.37	2.51	44.71	0.17	0.66	1.35	76.42	35.35	1.62
Q3	5.21	2.52	34.50	0.16	0.61	1.13	68.50	31.23	0.97
Q4	5.14	1.69	63.51	0.21	1.16	1.54	90.79	43.13	0.97
Q5	5.05	2.85	75.57	0.17	1.12	1.72	118.29	44.19	1.51
Q6	5.31	2.61	53.93	0.14	0.78	1.04	84.51	31.23	1.34
C1	5.96	2.38	42.18	0.27	1.46	1.06	76.42	33.10	1.78
C2	4.82	3.04	93.48	0.19	0.64	1.01	102.75	37.69	1.46
C3	4.17	8.28	45.16	0.26	1.99	2.08	196.67	48.30	1.81
C4	4.32	9.71	35.56	0.22	2.21	1.46	164.10	64.02	0.87
C5	4.58	2.82	93.48	0.17	1.08	1.57	120.43	46.56	0.49
J1	4.66	3.81	126.26	0.28	1.62	2.10	250.19	54.73	1.49
J2	4.57	4.76	63.82	0.19	0.97	1.36	419.77	44.18	1.02
J3	4.37	4.80	44.48	0.18	0.98	1.24	135.82	50.28	1.64
J4	5.05	2.46	72.28	0.31	1.25	1.12	111.29	41.06	1.37
B1	4.82	2.69	74.44	0.17	1.86	1.51	126.13	49.71	1.69
B2	4.81	2.90	56.15	0.18	1.36	1.38	179.45	41.10	0.82
B3	5.00	4.43	145.07	0.22	1.36	2.20	205.19	65.73	1.07
D1	5.01	3.68	90.37	0.22	1.09	1.70	143.80	54.99	1.22
D2	4.82	4.71	118.48	0.21	0.69	1.38	147.95	44.92	0.50

Abbreviations: A‐K, Available potassium; A‐N, Alkali‐hydrolyzed nitrogen; A‐P, Available phosphorus; S, Total amount water‐soluble salt; SOM, Soil organic matter; T‐K, Total potassium; T‐N, Total nitrogen; T‐P, Total phosphorus.

### 2.3 Data Processing and Analysis

#### 2.3.1 Important Values and Ecological Niche Calculations

Primarily, we wanted to determine the dominant position of each species in the shrub layer. For this, the average importance value (*IV*%) of all species in each of the 40 sample plots was used to screen the dominant species in the shrub layer. The importance values of all species in the shrub layer and the importance values of the dominant species used for analysis can be found in Appendix S1 and Appendix S2. The calculation formula used is as follows (Chen et al. 2021):

IV% = (relative abundance + relative frequency + relative coverage) / 3

$$\text{Relative abundance}=\left(\text{the abundance of}\;a\;\text{certain species in the quadrat}/\text{the}\;\mathrm{sum}\;\text{of the abundance of}\;\mathrm{all}\;\text{species}\right)\times 100\%$$

$$\text{Relative frequency}=\left(\text{frequency of}\;a\;\text{certain species in the sample}/\text{total frequency of}\;\mathrm{all}\;\text{species}\right)\times 100\%$$

$$\text{Relative coverage}=\left(\text{the coverage of}\;a\;\text{certain species in the sample}/\text{the total coverage of}\;\mathrm{all}\;\text{species}\right)\times 100\%$$

Niche and interspecific association analyses were conducted for dominant species with *IV*% > 1 in the shrub layer, using each site (or each environmental gradient) and each species importance value as different resource levels and species resource utilization states, respectively (Chen et al. 2021; Zhang, Zhao, and Luo 2018; Zheng et al. 2015). Niche width was calculated using the Shannon index (*B*_S_), and niche overlap was determined using the Pianka niche overlap index (*O*_*ik*_) (Liu et al. 2020). The calculation formula used is as follows:

Shannon's niche width (*B*_S_):

$$B_{S}=-\sum_{j=1}^{r}P_{\mathrm{ij}}\;\ln\left(P_{\mathrm{ij}}\right)$$

The Pianka niche overlap index (*O*_*ik*_):

$$O_{\mathrm{ik}}=\sum_{j=1}^{r}P_{\mathrm{ij}}P_{\mathrm{kj}}/\sqrt{\sum_{j=1}^{r}{\left(P_{\mathrm{ij}}\right)}^{2}\sum_{j=1}^{r}{\left(P_{\mathrm{kj}}\right)}^{2}}$$

In the formula, *P*_*ij*_ *= n*_*ij*_/*N*_*i*_, *P*_*kj*_ *= n*_*kj*_/*N*_*k*_, Herein, *n*_*ij*_ and *n*_*kj*_, respectively, represent the *IV*% of species *i* and species *k* at resource position *j*. *N*_*i*_ and *N*_*k*_, respectively, represent the sum of the *IV*% of the species *i* and species *k* at all resource positions. *P*_*ij*_ and *P*_*kj*_, respectively, represent the proportion of the IV% of species *i* and species *k* at resource position *j* as the sum of the *IV*% of that species at all resource positions; *r* is the total number of the plots. *O*_*ik*_ is the niche overlap index of species *i* and *k*, with the value domain of [0, 1], whose larger value indicates a higher degree of niche overlap (Liu et al. 2020).

#### 2.3.2 Phylogenetic Analysis and Nearest Taxon Index Calculation

We used the species, genus, and family lists of dominant species and the “V. PhyloMaker 2” package of R 4. 4. 1 to draw the phylogenetic tree, and the dominant species multiplicity (Appendix S3) and the “picante” package to calculate the net relatedness index (*NRI*) (Jin and Qian 2023). This index represents the standardized effect sizes of the mean phylogenetic distance (MPD) (Webb et al. 2002). The NRI was calculated as follow:

$$\mathrm{NRI}=\frac{{\mathrm{MPD}}_{\mathrm{obs}}-\text{mean}\;\left({\mathrm{MPD}}_{\mathrm{rand}}\right)}{\mathrm{SD}\;\left({\mathrm{MPD}}_{\mathrm{rand}}\right)}$$

*MPD*_*obs*_ denotes mean phylogenetic distance, mean (*MPD*_*rand*_) denotes the mean of the mean phylogenetic distances simulated randomly by the software 999 times, and SD is the standard deviation.

#### 2.3.3 Overall Association Test

The overall association was determined using variance ratio method (*VR*) (Schluter 1984) and test its significance by statistic *W*. The calculation formula used is as follows:

$$\mathrm{VR}=\frac{S_{T}^{2}}{\delta_{T}^{2}}=\frac{\frac{1}{N}\sum_{i=1}^{N}{\left(T_{j}-t\right)}^{2}}{\sum_{i=1}^{S}\frac{n_{i}}{N}\left(1-\frac{n_{i}}{N}\right)}$$

W=N×VR

In the formula, $S_{T}^{2}$_and_ $\delta_{T}^{2}$ are the variance of the number of species in all plots and the variance of the frequency of occurrence of all species, respectively; *N* is the total number of plots; *T*_*j*_ is the number of species in the plots *j*; *t* is the average of the number of species in each plot; *S* is the total number of species to be tested; and *n*_*i*_ is the number of plots where species *i* occurs. *VR* > 1 indicates a positive interspecific association, *VR* < 1 indicates a negative interspecific association, and *VR* = 1 indicates no association between a pair of species (Rousset and Lepart 2000). If $\chi_{0.95\left(N\right)}^{2}<W<\chi_{0.05\left(N\right)}^{2}$, the overall association between species is not significant (*p* > 0.05), and otherwise, the association is significant (*p* < 0.05) (Zhang 2011).

#### 2.3.4 Interspecific Associations and Interspecific Correlation Analyses

Because this study was based on non‐continuous sampling, the interspecific association was corrected by Yates continuous correction formula chi‐square statistics for qualitative study (Zhang 2011). Before the chi‐square test, data conversion was performed according to the selected dominant species, 0 means the species does not exist in the sample, and a value of 1 means the species exists. Conversion data are included in a table of 2 × 2 columns, and the values of *a*, *b*, *c*, and *d* were calculated. The calculation formula used is as follows:

$$\chi^{2}=\frac{N{\left(\left|\mathrm{ad}-\mathrm{bc}\right|-0.5N\right)}^{2}}{\left(a+b\right)\left(b+d\right)\left(c+d\right)\left(a+c\right)}$$

In the formula, *N* is the number of plots, *a* and *d* are the number of plots in which two species occur alone, and *b* and *c* are the number of plots where two species appear at the same time and do not appear at the same time, respectively.

The interspecific correlation chi‐square test only qualitatively describes whether a relationship between species are significant, whereas Pearson's correlation coefficient test and Spearman's rank correlation coefficient tests reflect clearly reflect the linear relationship between species (Jian et al. 2009). The two correlation coefficient tests can effectively complement and refine the correlation of other unrelated species pairs, the strength of connection of individual species pairs, and the differences in interspecific associations that were not accurately detected by the chi‐square test, through quantitative data. Therefore, in this study, quantitative correlations among dominant species were analyzed using the abundance of dominant species in the shrub layer as a quantitative indicator for Pearson's correlation coefficient test and Spearman's rank correlation coefficient test (Xu et al. 2016). The calculation formula used is as follows:

Pearson's correlation coefficients:

$$r_{p}\left(i,k\right)=\frac{\sum_{j=1}^{N}\left(x_{\mathrm{ij}}-{\bar{x}}_{i}\right)\left(x_{\mathrm{kj}}-{\bar{x}}_{k}\right)}{\sqrt{\sum_{j=1}^{N}{\left(x_{\mathrm{ij}}-{\bar{x}}_{i}\right)}^{2}\sum_{j=1}^{N}{\left(x_{\mathrm{kj}}-{\bar{x}}_{k}\right)}^{2}}}$$

Spearman's rank correlation coefficients:

$$r_{s}\left(i,k\right)=1-\frac{6\sum_{j=1}^{N}{\left(x_{\mathrm{ij}}-x_{\mathrm{kj}}\right)}^{2}}{N^{3}-N}$$

In the formula, *xij* and *xkj* are abundance values of species *i* and *k* in the resource bit *j*, respectively, and ${\bar{x}}_{i}$ and ${\bar{x}}_{k}$ are the means of species abundance for species *i* and *k* in all plots, respectively. The range of values for the two correlation coefficient tests is [−1, 1], with positive values indicating a positive correlation between the abundance of species, negative values being negative correlation, and 0 meaning no correlation. For information on calculating niche breadth, niche overlap, and the three significance tests, as well as the software packages used, please see Appendix S4.

#### 2.3.5 Environmental Interpretation

To better explain the environmental factors that lead to species coexistence and competition, we used the Canoco 5.0 software to conduct detrending correspondence analysis (DCA) on the relative importance values of dominant species. Afterward, we used a forward selection method of redundancy analysis (RDA). And Monte Carlo test (499 cycles) was used to screen the environmental factors with significant impact (*p* < 0.05) and then studied the impact of environmental factors on species distribution (Xiao, Zhong, et al. 2023).

We used regression analysis to further explore the effects of environmental factors on species distributions and the correlations between environmental factors. We chose the following 11 equations to fit for the regression analysis: linear function, quadratic function, cubic function, composite function *y* = *ab* ^*x*^, power function *y* = *ax* ^*b*^, S‐curve *y* = e^*a*+*b*/*x*^, growth function *y* = e^*a*+*bx*^, exponential function *y* = *a*e^*bx*^, logarithmic function *y* = *a* + *b*ln(*x*), inverse function *y* = *a* + *b*/*x*, and logistic function *y* = 1/(1/*c* + *ab* ^*x*^) (*a*, *b*, and *c* are parameters to be estimated). For regression analysis, we used box plots, Probability–Probability Plot (P–P Plots), and Quantile–Quantile Plot (Q–Q Plot) to test the data for anomalies, and then, the outliers are either replaced (by averaging all the y‐values corresponding to the same level of *x*‐values) or eliminated before proceeding with the regression analysis (Rahm and Hong 2000). In order to explore whether there are environmental differences between islands, we used principal component analysis (PCA) (Greenacre et al. 2022; Ringnér 2008) to rank the soil factors of the 40 sample plots and used the PC (principal components) axis values with the island isolation factor (the distance to the nearest island and the distance to the mainland) and climate factor to make regression analysis. If the explanation rate of the first three main eigenvectors is higher than 40%, it indicates that the ranking result is acceptable (Gauch 1989).

## 3 Results and Analysis

### 3.1 Important Value Characteristics of Dominant Species in the Shrub Layer

We identified 171 species of plants in the shrub layer on the island, of 23 species had an average importance value > 1 (Chen et al. 2021) and were considered to be the dominant species of the shrub layer. These 23 species can be found, sorted by importance value, in Table 3. The sum of the *IV*% of 23 species accounted for 70.43% of the total importance value of the shrub layer, indicating that these 23 species were important components of the community. Among them, *H. heptaphyllum* had the highest importance value and was the most dominant species in the shrub layer.

**TABLE 3:** Importance and niche breadth values of dominant species in shrub layer of *Pinus massoniana* community in 6 islands of the Sandu Gulf.

Abbreviation	Species (variety)	IV (%)	Niche width (*B*_S_)			
			Overall level	pH gradient	T‐K gradient	A‐K gradient
*Hh*	*Heptapleurum heptaphyllum*	7.63	3.23	2.22	2.02	2.14
*Lr*	*Litsea rotundifolia* var. *oblongifolia*	6.24	3.45	2.25	2.30	2.34
*Ss*	*Symplocos sumuntia*	5.67	3.47	2.26	2.32	2.28
*Rt*	*Rhodomyrtus tomentosa*	5.58	2.93	1.98	2.00	1.80
*Mp*1	*Mussaenda pubescens*	4.88	3.40	2.33	2.21	2.25
*Sc*	*Smilax china*	3.99	3.55	2.36	2.37	2.31
*Gj*	*Gardenia jasminoides*	3.88	3.43	2.24	2.25	2.31
*Am*	*Adinandra millettii*	3.68	3.26	2.09	2.25	2.17
*Pa*1	*Psychotria asiatica*	3.21	3.03	2.13	2.13	2.02
*Mm*	*Melastoma malabathricum*	3.08	2.98	2.07	2.12	2.09
*Sh*	*Syzygium hancei*	2.82	2.70	1.68	1.63	2.04
*Lc*	*Loropetalum chinense*	2.73	2.82	2.13	1.97	1.92
*Ip*	*Ilex pubescens*	2.42	3.50	2.31	2.35	2.30
*Mp*2	*Melicope pteleifolia*	2.16	2.94	2.00	2.10	2.04
*Ia*	*Ilex asprella*	1.83	2.71	1.83	2.01	2.02
*Ts*	*Toxicodendron succedaneum*	1.74	3.32	2.29	2.20	2.32
*Pa*2	*Pseudosasa amabilis*	1.57	2.02	1.48	1.83	1.48
*En*	*Eurya nitida*	1.45	3.02	2.17	2.18	2.09
*Sl*	*Smilax lanceifolia*	1.35	1.95	1.80	1.55	1.21
*Rc*	*Rubus corchorifolius*	1.21	2.67	1.88	1.76	2.09
*Sg*	*Smilax glabra*	1.19	3.20	2.18	2.17	2.23
*It*	*Ilex triflora*	1.06	3.03	2.24	1.87	2.18
*As*	*Alyxia sinensis*	1.05	2.92	2.02	1.99	2.13

### 3.2 Phylogenetic Analysis of Dominant Species

**FIGURE 2:**
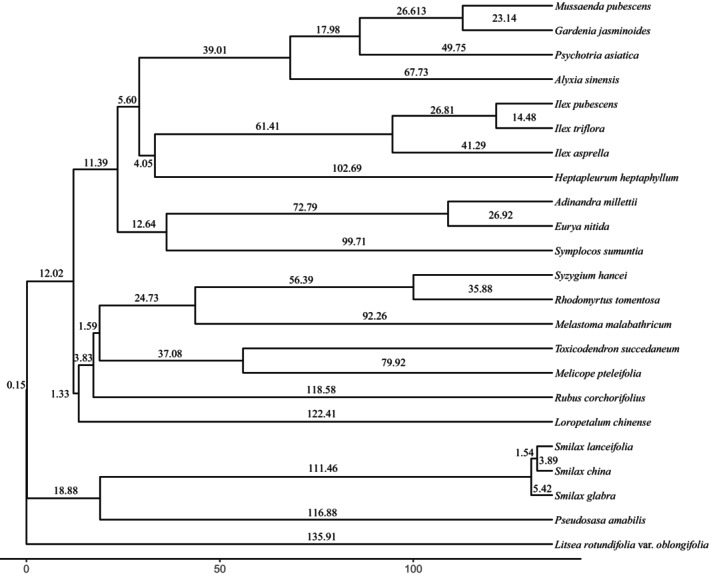
Phylogenetic tree of dominant species.

The phylogenetic tree of 23 dominant species is shown in Figure 2. The NRI results from 40 sample plots indicate that the community as a whole exhibits a non‐significant aggregation, with a positive to negative ratio of 2.08 (27:13) in the NRI.

### 3.3 Relationship Between the Importance of Dominant Species and Environmental Factors and Correlation Between Environmental Factors

**FIGURE 3:**
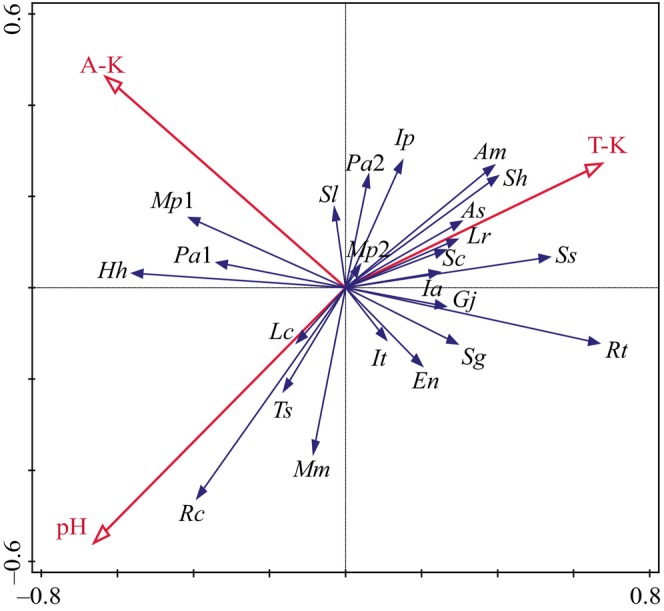
Redundancy analysis (RDA) analysis diagram of dominant species distribution with environmental factor in shrub layer. The species abbreviation are the same as in Table 3.

Relative importance values of 23 dominant plant species of shrub layer in 40 sample sites, environmental data, including 9 soil factors in Table 2, 5 topographic factors (elevation, slope aspect, slope, topographic position, distance from coastline), 2 island isolation factors (distance from nearest island and distance from mainland), 3 climatic factors and crown density, were analyzed by using redundancy analysis (Figure 3). Three environmental factors that significantly (*p* < 0.05) affected the important values of dominant species were selected, namely soil total potassium (T‐K, *p* = 0.006), pH (*p* = 0.02), and available potassium (A‐K, *p* = 0.03). Among them, soil T‐K content was positively correlated with the importance values of *Syzygium hancei*, *A. millettii*, *Alyxia sinensis*, *L. rotundifolia* var. *oblongifolia*, *Smilax china* , and other species, indicating that these species tended to favor habitats with higher T‐K content. T‐K content was negatively correlated with the importance values of *Rubus corchorifolius*, *T. succedaneum* , *Melastoma malabathricum* , *Loropetalum chinense*, and other species, indicating that these species are poorly adapted to habitats with high T‐K content. In contrast, pH was positively correlated with the importance values of *R. corchorifolius*, *T. succedaneum* , *M. malabathricum* , *L. chinense* , and other species, suggesting that these species fare poorly in low pH soils. pH was negatively correlated with the importance values of *S. hancei*, *A. millettii*, *A. sinensis* , *L. rotundifolia* var. *oblongifolia*, *S. china* , and others, suggesting that these species tend to favor habitats with acidic soils. Available potassium was positively correlated with the importance values of *M. pubescens* , *P. asiatica* , *H. heptaphyllum*, *S. lanceifolia*, and other species, indicating that these species tend to gravitate to habitats with higher A‐K content. A‐K was negatively correlated with the importance values of *R. tomentosa* , *Smilax glabra*, *Eurya nitida* , *G. jasminoides,* and other species, indicating that these species are not adapted to habitats with high levels of A‐K. These three environmental factors, namely soil T‐K, pH, and A‐K, together explained 24.9% of the *IV*% of the dominant species in the shrub layer, indicating that unknown factors that affected the *IV*% of the dominant species in the shrub layer of the communities studied accounted for 75.1%.

**FIGURE 4:**
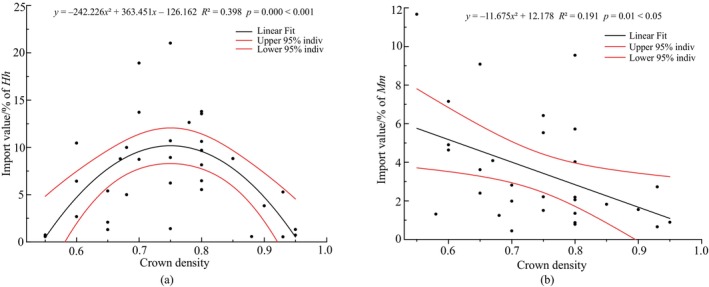
The regression between community crown density with important value of *Heptapleurum heptaphyllum* (a) and *Melastoma malabathricum* (b) in shrub layer.

Among the 23 dominant plants in the shrub layer, 20 plants showed neutral tlight tolerance such that the overall response to light (crown density) was insignificant. *H. heptaphyllum* was identified as tree species with a semi‐positive response to light, while *M. malabathricum* and *L. rotundifolia* var. *oblongifolia* had strongly positive responses. Scatter plots showing the relationship between the importance values of these three plants in 40 plots and crown density are displayed in Figure 4 (In the regression analysis, we first excluded 4 plots (S2, S6, S8, and S18) that were not distributed with *H. heptaphyllum* and 10 plots (S1, S4, S6, S9, S11, S19, S20, J4, B1, and B3) that did not have distribution of *M. malabathricum* . The outliers Q4 and D1 from Figure 4a and outlier Q3 from Figure 4b were then replaced to obtain the new Q4 (0.8, 9.69), D1 (0.85, 8.81), and Q3 (0.67, 22.21)). There was a significant (*p* < 0.05) quadratic function relationship between the importance value of *H. heptaphyllum* in the shrub layer and crown density. And the important value of *M. malabathricum* in the shrub layer was significantly (*p* < 0.05) negatively related to crown density. The positive plant *L. rotundifolia* var. *oblongifolia* has some shade tolerance. It grew poorly in shade, though the plant still survived. As a consequence, the results of the fit between its importance value in the shrub layer and the community depression were not significant (*p* > 0.05) and therefore not plotted.

**FIGURE 5:**
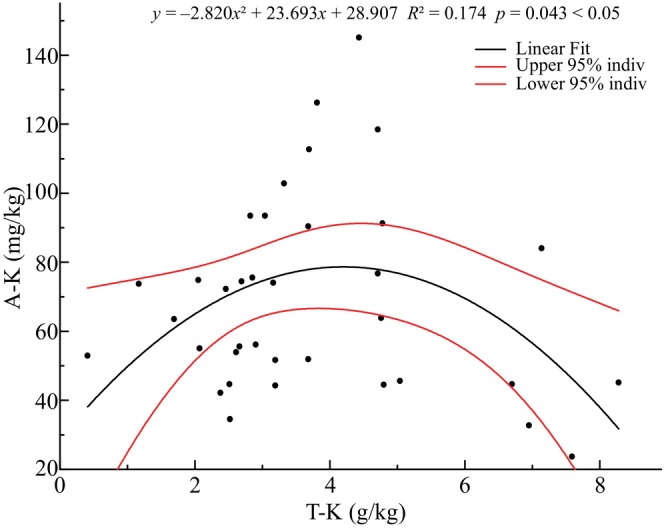
Analysis of the correlation between A‐K and T‐K.

To understand the correlation between soil available potassium (A‐K) content and soil total potassium (T‐K) content, curves were fitted to the two potassium measures of soil, and the results are shown in Figure 5 (Since T‐K and A‐K could not find similar points to be replaced, we directly eliminated four outliers: S9, S14, S20, and C4). The quadratic relationship between A‐K and T‐K was significant (*p* < 0.05), indicating that the A‐K content did not always increase with the T‐K content, and the A‐K content decreased in plots with higher T‐K content.

**FIGURE 6:**
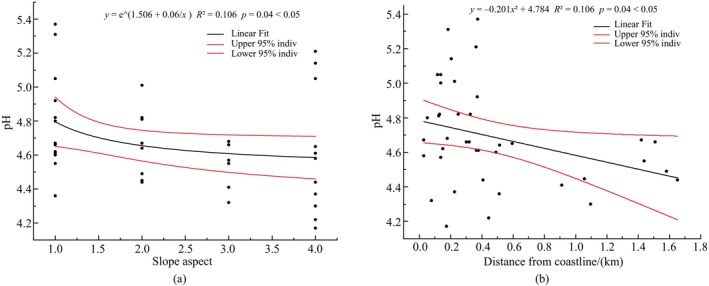
Analysis of the correlation between soil pH with slope aspect (a) and distance from coastline (b). 1: Adret; 2: Semi‐adret; 3: Semi‐ubac; 4: Ubac.

To understand the relationship between three soil factors (soil pH, T‐K, and A‐K) that significantly affect the *IV*% of the dominant species in the shrub layer and the topographic factor and crown density, correlation analyses were conducted on these factors and the results are shown in Figure 6 (We replaced the outliers C1 and B3 in Figure 6a and outlier C1 in Figure 6b to obtain the new C1(1, 4.80) and B3(1, 4.55)). There was a significant (*p* < 0.05) correlation between soil pH and slope direction, as well as between pH and the distance from the shoreline. That is, soil pH decreased significantly with increasing distance from the coastline and slope direction as we moved from adret to ubac.

**FIGURE 7:**
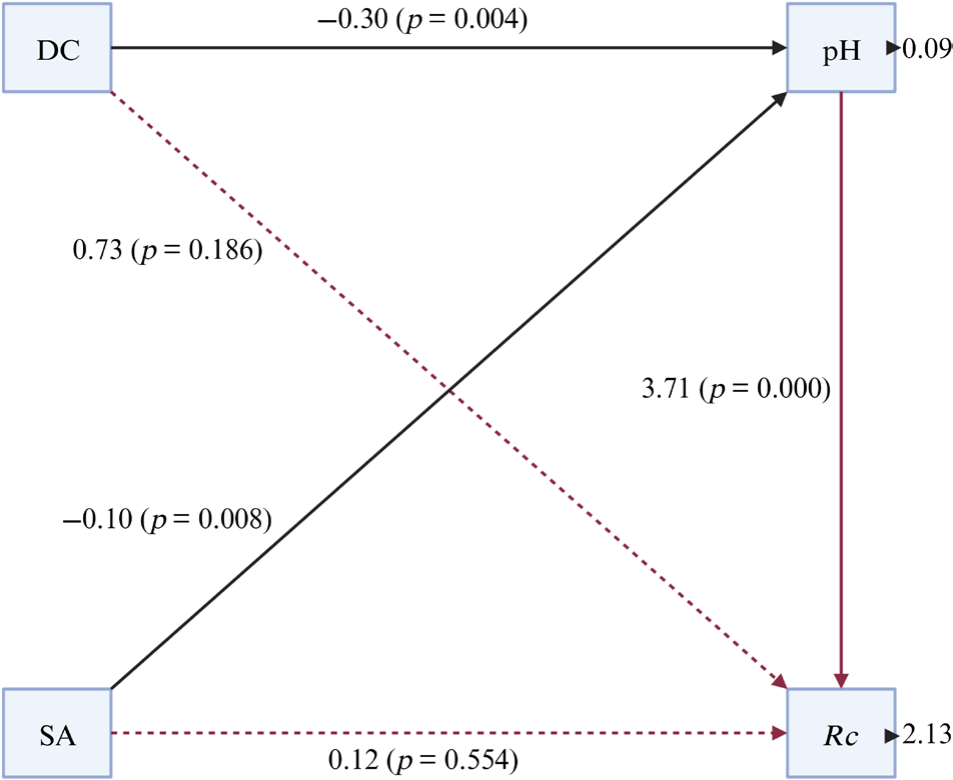
The results of the path analysis. SA: Slope aspect; DC, Distance from coastline; *Rc*, *Rubus corchorifolius*.

The strongest correlation was found between the importance value of *R. corchorifolius* and the pH value (Figure 3). In addition, slope direction and the distance from the coastline significantly affected the pH (Figure 6). For example, the important value of *R. corchorifolius* in the shrub layer was used to analyze the relationship between its important value with the pH, the slope direction, and the distance from the coastline. The results are shown in Figure 7. The results show that pH directly affected the magnitude of importance values associated with *R. corchorifolius*. The two showed a significant (*p* < 0.001) and positive correlation. But the effect of slope aspect and distance from the coastline on the importance value of *R. corchorifolius* was not significant (*p* > 0.05). The two affected the magnitude of important values of *R. corchorifolius* indirectly mainly by directly affecting the magnitude of pH (slope aspect and distance from the coastline showed a significant negative correlation with pH, *p* < 0.05).

To explore the effects of island isolation and climate on soil factors, we performed PCA ranking of the nine soil indicators. The results of the interpretation of the different axes of PCA ordination are shown in Table 4; Axis 1 (PC1) explained 31.66% of all the soil factors, and the first two axes explained a total of 50.49% > 40%, which indicates that the results of the ordination are acceptable. Interpretation rates of the nine soil indicators in different axes are shown in Table 5, with PC1 mainly reflecting the interpretation rates of T‐K, T‐P, N and SOM, and PC2 mainly reflecting the interpretation rates of pH.

**TABLE 4:** Importance of components.

Components	PC1	PC2	PC3	PC4	PC5	PC6	PC7	PC8	PC9
Eigenvalue	2.8498	1.6945	1.1759	1.0769	0.9883	0.50651	0.33796	0.21273	0.15737
Proportion Explained	0.3166	0.1883	0.1307	0.1197	0.1098	0.05628	0.03755	0.02364	0.01749
Cumulative Proportion	0.3166	0.5049	0.6356	0.7552	0.865	0.92133	0.95888	0.98251	1

**TABLE 5:** Species scores.

Components	PC1	PC2	PC3	PC4	PC5	PC6
pH	0.6963	−1.11282	0.25221	−0.09213	0.05576	−0.30912
T‐K	−0.8884	0.62512	0.66188	0.35501	0.23021	0.16463
A‐K	−0.4611	−0.67616	−1.03174	0.20183	−0.18287	0.22606
T‐P	−0.8078	−0.65273	0.53686	−0.59474	0.22	−0.36453
A‐P	−0.1935	0.44651	−0.15939	−1.26375	−0.34831	0.2359
T‐N	−1.1942	−0.55082	−0.04692	−0.02899	−0.21918	0.13926
A‐N	−0.8630	0.60662	−0.51756	0.10906	−0.22443	−0.78594
SOM	−1.2449	−0.36935	0.20690	0.19339	0.01625	0.23314
S	0.1868	−0.08824	0.50516	0.25624	−1.30362	−0.02561

*Note:* Positive and negative signs indicate direction. The larger the numerical value, the higher the interpretation rate.

**FIGURE 8:**
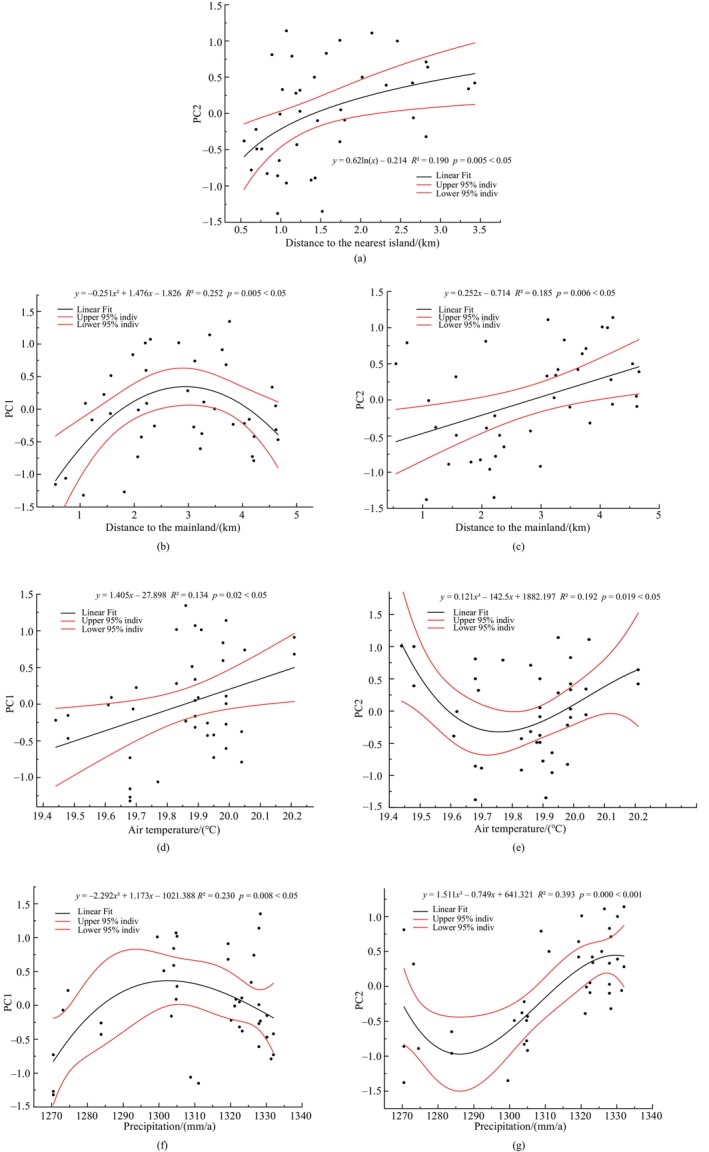
Regression analysis of PC1 and PC2 with isolation (distance to the nearest island (a) and mainland (b, c)) and climate factors (air temperature (d, e) and precipitation (f, g)).

We chose site scores (weighted sums of species scores) corresponding to PC1 and PC2 for regression analysis with isolation and climate factors, respectively. (We excluded seven data with wind speed > 1.6 m/s from the regression analysis of PC1 and PC2 with wind speed.) Plots with significant results are shown in Figure 8. Figure 8a,c shows a significant (*p* < 0.05) positive correlation between PC2 and distance from the mainland and nearest island. Combined with Table 5, it shows that the pH gradually decreased with increasing isolation. As can be seen in Figure 8b, there is a significant (*p* < 0.05) quadratic relationship between PC1 and distance from the mainland. The results show that T‐K, T‐P, A‐N, and SOM had a tendency of decreasing and then increasing with increasing isolation. Similarly, as shown in Figure 8d,e with the increase of temperature, T‐K, T‐P, N, and SOM gradually decreased, and pH showed a tendency of increasing and then decreasing. From Figure 8f,g, it can be seen that with increasing precipitation, T‐K, T‐P, N, and SOM showed a trend of decreasing and then increasing, and pH showed a trend of increasing and then decreasing. Combined with the results in Figures 3, 4, 5, 6, 7, it shows that although isolation and climate did not directly affect the distribution of dominant species in the shrub layer of the *P. massoniana* forest, they can indirectly affect it by directly affecting soil pH and other factors. As can be seen, there are many factors affecting the pH, which also leads to the small value of *R* ^2^ in the results in Figures 6 and 8.

### 3.4 Ecological Niche Characteristics of the Dominant Species in the Shrub Layer

To understand the effects of soil pH, total potassium, and available potassium on the niche width characteristics of the dominant species in the shrub layer, these three soil factors were divided into 11 gradient levels (Table 6), respectively. And the mean values of plant species importance values were calculated for each gradient level and the overall level (40 sample plots), from which the niche width was calculated (Zhang, Zhao, and Luo 2018). Appendices S5–7 present the importance values of available potassium, pH, and total potassium at different gradient levels. As shown in Table 3, the ranges of *B*_S_ indices for the overall level, pH gradient, T‐K gradient, and A‐K gradient were 1.95–3.55, 1.48–2.36, 1.55–2.37, and 1.21–2.34, respectively. *S. china* had the largest *B*_S_ index at the overall level, pH gradient and T‐K gradient, and *L. rotundifolia* var. *oblongifolia* had the largest *B*_S_ index at the A‐K gradient. *Smilax lanceifolia* had the smallest *B*_S_ index at the overall level, T‐K gradient and A‐K gradient, and *Pseudosasa amabilis* had the smallest *B*_S_ index at the pH gradient.

**TABLE 6:** Classification of pH, total potassium and available potassium gradient levels.

Gradient level	pH	T‐K/(g/kg)	A‐K/(mg/kg)
1	< 4.3	< 1.0	< 30
2	4.3~4.4	1.0~2.0	30~40
3	4.4~4.5	2.0~2.5	40~50
4	4.5~4.6	2.5~3.0	50~60
5	4.6~4.7	3.0~3.5	60~70
6	4.8~4.9	3.5~4.0	70~80
7	4.9~5.0	4.4~5.1	80~90
8	5.0~5.1	6.5~7.0	90~100
9	5.1~5.3	7.1~7.6	102~113
10	5.3~5.4	8.2~8.9	118~127
11	> 5.4	9.0~9.9	> 130

According to Table 7, the niche overlap value index (*O*_*ik*_) of the main dominant species of the shrub layer of *P. massoniana* communities studied was between 0.02 and 0.83. Among them, there were 87 species pairs with *O*_*ik*_ ≥ 0.5, accounting for 34.39% of the total number of pairs, and 166 species pairs with *O*_*ik*_ < 0.5, accounting for 65.61% of the total number of pairs. In total, 256 species pairs had an average ecological niche overlap value of 0.42, and all of them showed overlap.

**TABLE 7:** The niche overlap index of dominant species in shrub layer of *Pinus massoniana* community in six islands on the Sandu Gulf.

Species abbreviation	*Hh*	*Lr*	*Ss*	*Rt*	*Mp*1	*Sc*	*Gj*	*Am*	*Pa*1	*Mm*	*Sh*	*Lc*	*Ip*	*Mp*2	*Ia*	*Ts*	*Pa*2	*En*	*Sl*	*Rc*	*Sg*	*It*
*Lr*	0.46																					
*Ss*	0.40	0.77																				
*Rt*	0.14	0.61	0.78																			
*Mp*1	0.70	0.51	0.53	0.25																		
*Sc*	0.45	0.78	0.83	0.66	0.68																	
*Gj*	0.43	0.69	0.80	0.74	0.58	0.76																
*Am*	0.27	0.69	0.82	0.72	0.50	0.82	0.71															
*Pa*1	0.64	0.49	0.47	0.19	0.53	0.43	0.52	0.38														
*Mm*	0.39	0.29	0.38	0.28	0.58	0.58	0.42	0.40	0.25													
*Sh*	0.17	0.48	0.54	0.43	0.28	0.38	0.29	0.51	0.20	0.16												
*Lc*	0.28	0.48	0.54	0.39	0.27	0.50	0.46	0.55	0.27	0.28	0.26											
*Ip*	0.52	0.78	0.76	0.56	0.72	0.76	0.67	0.70	0.52	0.41	0.43	0.42										
*Mp*2	0.39	0.53	0.55	0.22	0.45	0.48	0.52	0.44	0.58	0.22	0.37	0.31	0.46									
*Ia*	0.19	0.34	0.52	0.32	0.39	0.49	0.35	0.48	0.29	0.37	0.63	0.19	0.36	0.62								
*Ts*	0.70	0.49	0.53	0.32	0.74	0.65	0.53	0.39	0.51	0.55	0.31	0.36	0.56	0.42	0.47							
*Pa*2	0.15	0.33	0.40	0.32	0.32	0.51	0.42	0.38	0.16	0.19	0.07	0.12	0.42	0.27	0.13	0.20						
*En*	0.31	0.56	0.65	0.45	0.46	0.52	0.41	0.53	0.27	0.48	0.54	0.44	0.63	0.33	0.44	0.38	0.10					
*Sl*	0.18	0.24	0.22	0.73	0.22	0.22	0.21	0.16	0.18	0.07	0.30	0.07	0.24	0.47	0.20	0.18	0.06	0.13				
*Rc*	0.50	0.24	0.25	0.12	0.58	0.46	0.28	0.27	0.30	0.68	0.06	0.42	0.40	0.17	0.13	0.48	0.06	0.29	0.04			
*Sg*	0.41	0.56	0.66	0.69	0.52	0.71	0.65	0.62	0.30	0.54	0.42	0.30	0.69	0.24	0.40	0.63	0.33	0.42	0.14	0.41		
*It*	0.56	0.53	0.56	0.38	0.46	0.51	0.49	0.37	0.40	0.28	0.32	0.30	0.50	0.44	0.39	0.59	0.02	0.37	0.17	0.42	0.53	
*As*	0.28	0.56	0.49	0.41	0.35	0.51	0.38	0.49	0.24	0.25	0.37	0.26	0.56	0.34	0.30	0.38	0.11	0.43	0.39	0.12	0.53	0.37

### 3.5 Overall Association of the Predominant Species in the Shrub Layer

The variance ratio of the overall association, *VR* = 1.34 > 1 indicated a generally positive association between dominant species. After checking the table, the test statistic *W* = 53.62 was between $\chi_{0.95\left(40\right)}^{2}\left(26.51\right)<W<\chi_{0.05\left(40\right)}^{2}\left(55.76\right)$. Therefore, the overall association of the dominant species in shrub layer of the communities studied was not significant positive association.

### 3.6 Interspecific Association and Correlation Analysis of Dominant Species in the Shrub Layer

**FIGURE 9:**
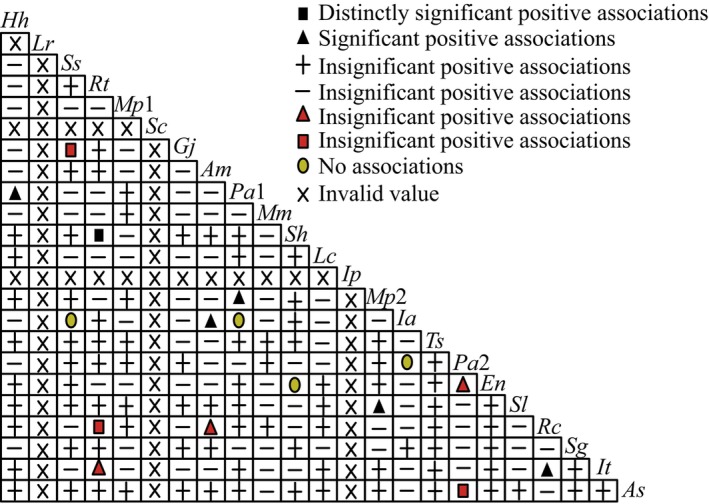
Semi‐matrix diagram of chi‐square test coefficients among predominant species in shrub layer of *Pinus massoniana* community in six islands on the Sandu Gulf.

The dominant species chi‐square test results (Figure 9 and Table 8) identified 94 pairs of species (49.47%) were positively associated with each other. The species pairs with extremely significant, significant, and insignificant positive associations and show 1, 5, and 88 positive associations, respectively, accounting for 0.53%, 2.63%, and 46.32% of the total effective log, respectively. Negative association species pairs showed 92 pairs for 48.42% of the total effective log. The species pairs showed 3, 3, and 86 with extremely significant, significant, and non‐significant negative associations, respectively, accounting for 1.58%, 1.58%, and 45.26% of the total log, respectively. The positive and negative association ratio was 1.02, indicating a small logarithmic difference between positive and negative correlation species and a slightly dominant positive correlation. A total of 178 pairs of non‐significantly associated and non‐associated species pairs were found, accounting for 93.68% of the total. The significance rate was only 6.32%, consistent with the overall association test, showing an insignificant positive correlation. This result showed that the interspecific relationships of dominant species in the shrub layer were relatively loose with an independent distribution pattern.

**TABLE 8:** The comparison of chi‐square text coefficients, Pearson's correlation coefficients, and Spearman's rank correlation coefficients among predominant species in shrub layer of *Pinus massoniana* community in six islands on the Sandu Gulf.

Test methods	Positive association				Negative association				No association
	Distinctly significant	Significant	Not significant	Sum	Distinctly significant	Significant	Not significant	Sum	
*χ* ^2^ test	1 (0.53)	5 (2.63)	88 (46.32)	94 (49.47)	3 (1.58)	3 (1.58)	86 (45.26)	92 (48.42)	4 (2.11)
Pearson's correlation	13 (5.14)	11 (4.35)	106 (41.90)	130 (51.38)	2 (0.79)	5 (1.98)	116 (45.85)	123 (48.62)	0 (0.00)
Spearman's rank correlation	16 (6.32)	16 (6.32)	112 (44.27)	144 (56.92)	14 (5.53)	8 (3.16)	87 (34.39)	109 (43.08)	0 (0.00)

**FIGURE 10:**
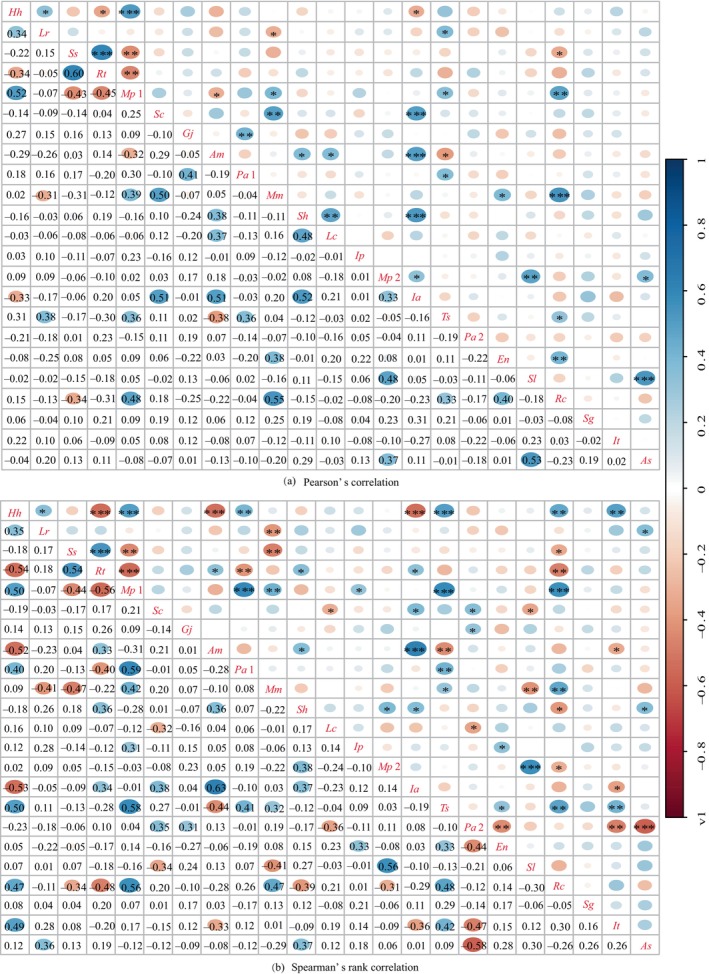
Semi‐matrix diagram of Pearson's correlation coefficients and Spearman's rank correlation coefficients among predominant species in shrub layer of *Pinus massoniana* community in six islands on the Sandu Gulf. *p < 0.05; **p < 0.01; ***p < 0.001.

Pearson's correlation test results of the dominant species (Figure 10 and Table 8) revealed that among the 253 species pairs, those with positive associations had 130 pairs, accounting for 51.38% of the total log. The species pairs had 13, 11, and 106 pairs with extremely significant, significant, and insignificant positive associations, respectively, representing 5.14%, 4.35%, and 41.90% of the total log numbers, respectively. Seed pairs with negative associations had 123 pairs accounting for 48.62% of the total log. The species pairs displayed extremely significant, significant, and non‐significant negative associations, respectively, accounting for 0.79%, 1.98%, and 45.85% of the total log.

The Spearman rank correlation test of the dominant species (Figure 10 and Table 8) revealed that among 253 species pairs, 144 species pairs were positively correlated, accounting for 56.92% of the total log. Species pairs with extremely significant, significant, and non‐significant positive associations were 16, 16, and 112 pairs, respectively, accounting for 6.32%, 6.32%, and 44.27% of the total log. Species pairs with negative associations had 109 pairs, accounting for 43.08% of the total log. The species pairs had 14, 8, and 87 pairs with extremely significant, significant, and non‐significant negative associations, respectively, accounting for 5.53%, 3.16%, and 34.39% of the total log, respectively.

The positive and negative association ratios for these two tests were and 1.06 and 1.32, respectively, indicating little difference between the logarithms of positive and negative correlation species, with slightly dominant positive correlation. The significance rate for these two tests was 12.25% and 21.34%, indicating that the interspecific relationships of dominant species in the shrub layer were relatively loose with an independent distribution pattern.

### 3.7 Regression Analysis of Phylogenetic Distance, Interspecies Association, and Ecological Niche Overlap

**FIGURE 11:**
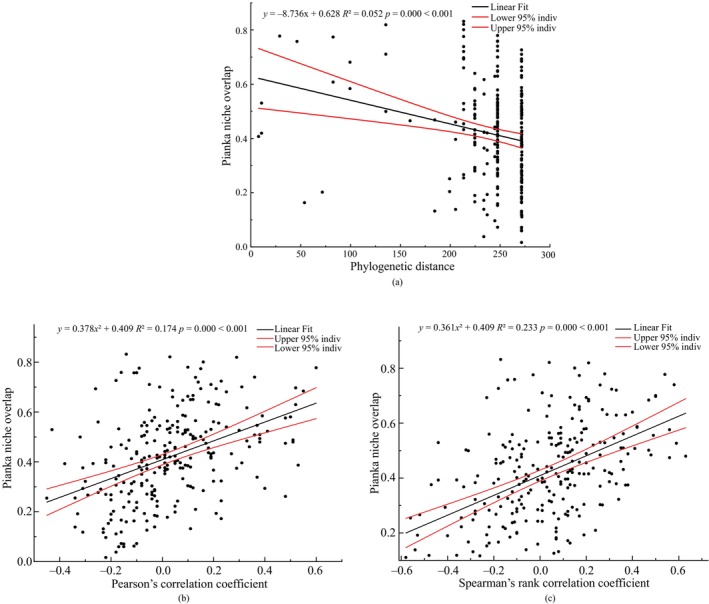
Regression analysis between phylogenetic distance (a), Pearson's correlation coefficients (b), Spearman's rank correlation coefficients (c), and niche overlap among predominant species in shrub layer of *Pinus massoniana* community in six islands in Sandu Gulf.

Phylogenetic distance, Pearson's correlation coefficient, and Spearman's rank correlation coefficient (*p* < 0.001) between the dominant species, as shown in Figure 11, indicated that closer (farther) phylogenetic distances and stronger positive (negative) connection between the species were related to a greater degree of niche overlap (smaller).

### 3.8 Ecological Species Group Division

Based on the RDA ranking results (Figure 3) combined with information from the chi‐square test, Pearson's correlation test, and Spearman's rank correlation test results, the dominant species in the shrub layer were roughly divided into three ecological species groups (Jiang and Zhang 2021). Ecological species group I: *R. tomentosa* (*Rt*), *A. millettii* (*Am*), *G. jasminoides* (*Gj*), *S. sumuntia* (*Ss*), *L. rotundifolia var. Oblongifolia* (*Lr*), *S. hancei* (*Sh*), *P. amabilis* (*Pa*2), *S. china* (*Sc*), *Ilex asprella* (*Ia*), *Ilex pubescens* (*Ip*), *T. succedaneum* (*Ts*), *L. chinense* (*Lc*), *A. sinensis* (*As*), *S. lanceifolia* (*Sl*), and *Melicope pteleifolia* (*Mp*2); this group has the largest number of individual plants, is better adapted to light, and is able to use the potassium in total potassium to supply plants with the required nutrients. Among them, many species, such as *A. millettii*, *R. tomentosa,* and *S. sumuntia*, are mainly distributed in areas far from the coastline with extremely acidic soils. We considered *R. corchorifolius* (*Rc*), *M. malabathricum* (*Mm*), *S. glabra* (*Sg*), *E. nitida* (*En*), and *Ilex triflora* (*It*) as a second ecological group, group II. The species in this group are light‐loving and intolerant of acidic strong soils, and are mainly distributed on adret slopes or in slightly acidic soil areas with low crown density and high light availability; these species do not have a high demand for potassium, and excessive soil potassium content is not conducive to the growth of these plants. Ecological species group III included *H. heptaphyllum* (*Hh*), *Psychotria asiatica* (*Pa*1), and *M. pubescens* (*Mp*1). Species in this group mainly use potassium from available potassium to supply nutrients, are not tolerant to strong acids, and are mainly found in slightly acidic soils.

## 4 Discussion

### 4.1 Important Value and Niche Breadth of Dominant Species in Shrub Layer

Niche breadth reflects the ecological adaptability of plant populations and their ability to use environmental resources (Yu et al. 2017). In this study, the niche breadths (as well as important values) of *S. china* , *S. sumuntia*, and *I. pubescens* under various horizontal gradients were larger, suggesting these species were broadly distributed across the islands and had a strong resource utilization ability and environmental adaptability. Conversely, the niche breadth of *S. china* and *P. amabilis* was narrow, suggesting that the spatial distribution of these species was not uniform, and that these species were predominantly restricted to specific habitats. They are easily eliminated in the succession of the communities. For example, in our study, *P. amabilis* only appeared within a pH range of 4.4~5.1, *S. hancei* mainly appeared when pH value dipped below 5.1, and *S. china* struggled to survive in habitats with excessively high or low potassium content.

Important value and niche breadth are two significant indicators with not exactly the same ecological significance, which jointly reflect the status and function of species in the community (Liu et al. 2020). While important value which mainly reflects the dominance of species is often positively correlated with niche breadth, niche breadth may be greatly affected by the distribution frequency. This was consistent with our understanding of dominance and the maintenance of diversity (Chen et al. 2021; Liu et al. 2020). In our study, *H. heptaphyllum* exhibited the largest important value and was the dominant species in the shrub layer of the *P. massoniana* community. However, its highest niche breadth just ranked ninth in each gradient. Similarly, *M. malabathricum* showed a large important value despite having a small niche breadth. This is predominantly due to the limited distribution area. The frequency of distribution is therefore reduced, so as the niche breadth. *H. heptaphyllum* can survive in full sun, half sun and half shade environment, growing best in the latter two environments and poorly under the first one, and cannot survive in the shade. Therefore, *H. heptaphyllum* in shrub layer mainly distributed in the habitat where the community canopy was about 0.7~0.8. Additionally, it struggled to survive in plots with low content of available potassium or total potassium. In contrast, *M. malabathricum* preferred light‐rich environments and mainly occupied in habitats with low canopy density. Many species also exhibited lower abundance in plots with low available potassium and total potassium content, resulting in an overall reduced niche breadth due to the constraint imposed by the minimum potassium requirement. On the other hand, certain species such as *S. glabra* , *T. succedaneum* , and *I. pubescens* had relatively small important values but exhibited high niche breadth. This can be attributed to their wide distribution and the production of micro fruit (fruit diameter ≤ 1 cm), which can be dispersed through various mechanisms such as autogamy, animal dispersal, and wind dispersal (He et al. 2022), thereby contributing to a large niche breadth.

### 4.2 Overall Environmental Analysis

The results of the redundancy analysis (RDA) screened out three soil environmental factors that significantly (*p* < 0.05) affected the *IV*% of the dominant species, namely total potassium (T‐K), pH, and available potassium (A‐K). While island isolation and climate were not significant. We believe this is likely because all six islands in the study area are geographically close to each other and are all inland islands that are less affected by speed of wind. Consequently, there is little difference in climate between islands and therefore no significant correlation with climate factors. Additionally, since the focus of this study was on dominant species rather than all species, and the dominant species were found to be relatively consistent across the six islands, there was no significant correlation with island isolation. However, island isolation and climate still influenced the distribution of plant communities indirectly by affecting soil pH and other soil chemical content. Xu et al. (2023) analyzed that plant diversity decreases as island isolation increases. The decrease in plant diversity may result in inadequate litter supply for microbial decomposition, leading to a decrease in soil organic matter (SOM), nitrogen (N), phosphorus (P), and potassium (K) content. And the accumulation of litter may lead to an increase in soil water content. According to Chen et al. (2003) and Reth, Reichstein, and Falge (2005), they observed that soil acidity in areas with high precipitation was stronger than that in arid areas. This consequently leads to a decrease in the pH of the soil. However, Gao, Wang, and Yu (2015) argued that with increasing distance from the mainland, the composition and structure of plant communities will become more simplified. Thereby, this makes more remote islands more vulnerable to plant invasions and the colonization and spread of animal and microbial species as reported by Jesse et al. (2018), Moser et al. (2018), and Pyšek et al. (2010). The colonization and spread of microorganisms promotes the decomposition of litter, and soil contents of N, P, K, and SOM may increase as a result. And organic matter can effectively regulate the content of nitrogen and effective phosphorus elements in the soil, while reducing the content of alkaline nitrogen and the possibility of soil salinization (Jing et al. 2016; Xiao, Zhong, et al. 2023). As a result, soil pH continue to decrease. Higher temperatures and water evaporation may increase pH. However, Allison and Treseder (2008) showed that increased temperatures inhibit microbial activity, resulting in decreased rates of decomposition of litter. According to Hong et al. (2022), higher temperatures prolong the leaf life of herbaceous plants and indirectly lead to herbaceous litter buildup and untimely decomposition. Soil moisture content may increase as a result. Thus, increasing temperature also leads to the decrease in pH, N, P, K, and SOM of soil. The effect of precipitation on soil composition is a more complex process. Hessel and Van Asch (2003) explored that precipitation may lead to runoff and soil erosion, which in turn reduces soil chemical content, whereas precipitation also increases plant biomass, accelerates litter fragmentation and leaching, and enhances soil microbial activity, which in turn promotes litter decomposition and elemental release (Santonja et al. 2018). It can be seen that there are a large number of factors affecting soil pH, N, P, K, etc., including abiotic factors (topography, climate, isolation, etc.) and biotic factors (plants, soil microorganisms, etc.). As shown in a study conducted by Dias, Cornelissen, and Berg (2017), litter decomposition is determined by a combination of plant species, climate, and other factors. This study (Figure 8) also confirms the above results.

Total potassium can be divided into water‐soluble potassium, non‐special adsorptive potassium, and special adsorptive potassium three forms of available potassium, as well as non‐exchangeable potassium (slowly available potassium) and mineral potassium (Jin 1993). The effective component of available and non‐exchangeable potassium is called effective potassium that can be absorbed and utilized by plants (Zhao et al. 2010). Potassium is an essential nutrient for plants, involved in the activation of plant enzymes, protein synthesis, and nutrient transport (Shen 1982). Furthermore, it can also promote the assimilation of leaves and the growth of branches (Xu et al. 2019). Potassium helps plants growth and development in the appropriate amounts, otherwise inhibits according to Xu and Deng (2023). Figure 9 showed that sample plots with high total potassium content had low available potassium content, indicating a high content of non‐exchangeable potassium and mineral potassium in these plots. It showed that species such as *S. hancei*, *A. millettii*, and *A. sinensis* can effectively use all the effective potassium content in total potassium, especially the effective component of the non‐exchangeable potassium, whereas species such as *M. pubescens* , *P. asiatica*, and *H. heptaphyllum* largely utilize the effective component of available potassium. The use of potassium varies largely among these species, resulting in a small niche overlap (Table 7).

Different plants exhibited varying degrees of adaptability to soil pH. Plants such as *A. millettii* and *S. hancei* preferred to live in habitats with higher pH than those of *R. corchorifolius* and *T. succedaneum* . The absorption of many elements by plants is weakened when pH is too low (Liu et al. 2018), which is not conducive to the growth of these plants. Figure 6 showed that the soil pH decreased significantly (*p* < 0.05) from the adret slope to the ubac slope, which could be attributed to the difference in the soil moisture content. Liu and Ma (2012) argued that the ubac slopes are cooler than the adret slopes, with higher soil moisture content. High moisture content leads to a decrease in pH. Soil pH decreased significantly (*p* < 0.05) with the increase of the distance from the sample plot to the coastline. This can be primarily attributed to the closer proximity to the coast, making the sample plot more susceptible to the influence of alkaline seawater, resulting in relatively high soil pH.

### 4.3 Phylogenetic Analysis, Niche Overlap, Interspecific Associations and Their Environmental Interpretation of Dominant Species in Shrub Layer

The phylogenetic distance and ecological niche overlap index exhibited a significant negative correlation (Figure 11a), indicating that species with closer genetic distances had higher ecological similarity in their resource requirements. However, the relatively small *R* ^2^ value can be attributed to the fact that as the community underwent succession, the growth and abundance of ecologically similar species increase. Consequently, the resources required by the plants cannot be fully satisfied, leading to intensified interspecific competition. This results in the gradual transition of closely related species from aggregation to dispersion within the community (Cui and Tie 2021), as observed in species pairs such as *A. millettii*‐ *E. nitida* (53.84, 0.16) and *S. hancei*‐ *R. tomentosa* (71.75, 0.2). Furthermore, these species pairs exhibited significantly different potassium element requirements and were distributed in different ecological species groups, leading to lower ecological niche overlap values. Similar conclusions could be drawn for species pairs with distant phylogenetic distances. For instance, species pairs with ecological niche overlap indices ≥ 0.8, such as *S. sumuntia*‐ *S. china* , *S. china* ‐ *G. jasminoides* , *S. china* ‐*A. millettii*, and *S. sumuntia*‐*A. millettii*, exhibited high ecological niche overlap despite their distant phylogenetic distances. This is primarily due to their close environmental requirements and distribution within the same ecological species groups. These findings indicate that species phylogenetic relationships, competition, and environmental heterogeneity all influence the magnitude of ecological niche overlap among plants.

Greater niche overlap suggests a more similar lifestyle and ecological demand for environmental resources among species, and possibly more intense competition (Li et al. 2014). In our study, we identified several species pairs with a niche overlap index ≥ 0.8. The large niche breadth of these species indicated high ecological similarity and the potential for fierce competition, particularly under conditions of resource scarcity. Species pairs with niche overlap index < 0.1 included *M. malabathricum* ‐*S. lanceifolia*, *S. hancei*‐ *P. amabilis* , *S. hancei*‐*R. corchorifolius*, *Loropetalum chinense*‐*S. lanceifolia*, *P. amabilis* ‐*S. lanceifolia*, *P. amabilis* ‐*R. corchorifolius*, *P. amabilis* ‐*I. triflora*, and *S. lanceifolia*‐*R. corchorifolius*. The niche breadth of these species was small suggesting a commensurately small level of competition. We did observe that several of these species had common pairings with a high niche overlap but low niche breadth, such as *M. malabathricum* ‐*R. corchorifolius* (0.68), *S. hancei*‐*I. asprella* (0.63), *Melicope pteleifolia*‐*I. asprella* (0.62), and *I. triflora‐I. pubescens* (0.5). The primary reason for the higher degree of niche overlap of *M. malabathricum* and *R. corchorifolius* is that they are both shrubs, and their life form and growth height are relatively close, and both of them are present in a soil with low T‐K, low A‐K, and high pH, whereas *I. asprella*, *S. hanceii*, and *M. pteleifolia* are present in a soil with high T‐K and low pH. These species pairs are then showed a fierce competition between themselves respectively with a great amount of niche overlap—largely because they are confined to the same local environment (i.e., low T‐K, low A‐K, and high pH in the case of *M. malabathricum* and *R. corchorifolius*). Although *I. triflora* and *I. pubescens* exhibited significant differences in potassium element requirements and were distributed in different ecological species groups, their close phylogenetic distance, similar life form, and growth habit resulted in a relatively high ecological niche overlap.

Interspecific association reflects the interaction between species and community dynamics, whereas the overall association reflects the process and stability of community succession (Jin et al. 2013). The community in the early succession is in an unstable stage with a low degree of association between species and a small positive–negative association ratio (Liu et al. 2020). Similarly, community stability is positively associated with the degree of association between species and the number of species pairs (Liu et al. 2020). When the community succession reached the climax, the species exhibited significant positive associations, with an increase in unrelated association pairs (Liu et al. 2020). According to the results of chi‐square test, Pearson's correlation test, and Spearman's rank correlation test, the interspecific association between species pairs of dominant species in the shrub layer from the communities studied was slightly dominated by a positive association. However, the significance of species pairs was low, with small positive and negative association ratios of the three tests (1.02, 1.06, and 1.32). And the overall association indicated an insignificant positive association, and the *NRI* exhibited a non‐significant aggregation, indicating that the community was still in the process of succession and was not yet at its most stable state. This aligns with our understanding of the *P. massoniana* communities along the Sandu Gulf (Xiao, Lai, et al. 2023; Xiao, Zhong, et al. 2023), indicating that they are predominantly in the stage of succession of coniferous and mixed coniferous forests and need to continue to move toward stable coexistence. At the same time, the shortage of habitat resources caused by the harsh environment of the island will also force species to gather in the same habitat, leading to a noticeable degree of niche overlap between species pairs. Furthermore, there were obvious competitive relationships in many pairs, and the dominance of positive association pairs and unrelated association pairs was weak.

Li et al. (2017) argued that positive interspecific association reflects the similarity of species utilization resources and the overlap of niches. A stronger positive association was related to a greater niche overlap (Liu et al. 2020). Many studies have also shown that species within the same ecological species group have similar ecological habits and habitat characteristics, and can utilize the same resources in the community (Tu et al. 2019). And they are interdependent on each other and coexist; thus, most of the species pairs within the group are positively associated. On the contrary, there are differences in the ecological habits and habitat characteristics of the species in different ecological species groups, and species are mutually exclusive (Tu et al. 2019). So the species pairs between groups are mostly are mostly negative association. As shown in the Figure 5, this study also reached a similar conclusion. According to Table 7, Figures 3 and 4 and the results of ecological species group division, we observed high levels of overlap in species pairs such as *H. heptaphyllum*‐ *M. pubescens* (0.70) and *S. sumuntia*‐ *R. tomentosa* (0.78), with a significant positive association relationship. The main reason is that these species pairs were in the same ecological species group and tend to live in similar habitats. For example, *H. heptaphyllum* and *M. pubescens* prefer habitats with high A‐K, and low T‐K, whereas *S. sumuntia* and *R. tomentosa* prefer habitats with high T‐K, low A‐K, and low pH. These results may suggest that these species‐pairs are in the process of co‐evolution (Chen et al. 2021). These results may also reflect positive interspecific association associated with plants that occupy different parts of the vertical habitat. *H. heptaphyllum* and *S. sumuntia* are arbor species, and *M. pubescens* and *R. tomentosa* are shrub species. Large differences exist in the growth height of these species, which can form a complementary habitat in the vertical direction (Zhou et al. 2015). This may contribute to the highly significant positive correlation in their abundance and corresponding high niche overlap.

Interspecific negative associations are largely affected by habitat differences and resource competition (Zhang, Zhao, and Luo 2018). In general, the former causes negative associations with low overlap species pair and the latter causes high. Among all species pairs with extremely significant negative linkage in this study, only *S. sumuntia*‐ *G. jasminoides* (0.80) had a high degree of niche overlap, whereas other species pairs, such as *R. tomentosa* ‐*R. corchorifolius* (0.12), *P. amabilis* ‐ *A. sinensis* (0.11), and *H. heptaphyllum*‐*A. millettii* (0.27), had a low degree of niche overlap. The RDA analysis results showed that the important values of *S. sumuntia* and *G. jasminoides* were positively correlated with T‐K, but exhibited negative connection caused by resource competition of T‐K, with high niche overlap of species pairs, whereas other species pairs were in different ecological species groups and tend to live in different and distinct soil (in terms of their potassium content and soil pH), leading to the differences of habitats. Thus, the niche overlap of negative connection of species pairs were low. However, there were also significant positive associations between species in different ecological species groups, such as significant positive associations among many species in ecological species groups II and III. The main reason is that the similarity of habitat preference existed between the plants of ecological species groups II and III. That is, there are similarities in habitat preferences, namely intolerance of strong acids mainly distributed in slightly acidic soil, which leads to a significant positive association.

## 5 Suggestions

From the results, the analysis of the effects of environmental factors is crucial for the classification of ecological species groups and the interpretation of interspecific connectivity results. Therefore, it is recommended to include the interpretation of environmental factors when conducting future interspecific association‐related studies. In the future, plants with large niche widths, such as *S. china* , *S. sumuntia*, and *L. rotundifolia* var. *oblongifolia*, should be selected as the main tree species to plant undergrowth shrubs when vegetation restoration and plantations construction is performed for relevant island communities. These species have strong ecological adaptability, can be widely distributed in the shrub layer of the communities studied, and belong to the dominant species of the shrub layer. At the same time, plants with a small niche overlap with these species should be selected for planting; for example, *S. lanceifolia* and *R. corchorifolius* can effectively reduce competition among species and maintain the stability of the community. We can also select species pairs with significant positive associations to plant, such as *H. heptaphyllum*‐ *M. pubescens* and *S. sumuntia*‐ *R. tomentosa* . These pairs of species have similar utilization of resources, and species pairs can tend to co‐evolve. Species pairs can form complementarities with habitats in the vertical direction, with less intense interspecific competition. In the presence of certain species of positive plants in the community, such as *H. heptaphyllum* and *M. malabathricum* , the gap area should be appropriately increased to provide sufficient light on these species. The soil nutrient content, such as potassium, should be monitored regularly, and potassium fertilizer should be properly applied to promote the growth and development of plants. On the adret slope and near the coast, plant species with weak resistance to slightly acidic soil, such as *A. millettii* and *S. hancei*, while in the ubac slope and far coast, plant species preferring acid soil, such as *R. corchorifolius* and *T. succedaneum* . In addition, reducing the crown density of the ubac slope plant community can enhance soil water evaporation and effectively improve soil pH. Since the factors affecting soil pH and chemical content include not only abiotic but also biotic factors (plants, animals, microorganisms, etc.), it is suggested that biotic factors should be included in the study in the future. The interaction between various factors was comprehensively discussed, the environmental adaptation mechanism of plants was explored, and a more comprehensive and reasonable plant allocation scheme was formulated, which could provide a reference for the restoration of future island vegetation and the optimization of island plantation structure.

## 6 Conclusions

After studying the niche, interspecific associations, stability of the dominant species in the shrub layer of the *P. massoniana* community in six islands of Sandu Gulf, Ningde, and the environmental factors affecting the important values of the dominant species in the shrub layer, we found that species such as *S. china* , *L. rotundifolia* var. *Oblongifolia*, *S. sumuntia*, and *I. pubescens* occupied a larger ecological niche width in the shrub layer and could better adapt to the ecological environment in six islands in Sandu Gulf. There was no absolute positive correlation between the niche width of the dominant species and the degree of niche overlap in the shrub layer of the communities studied. This was also mainly influenced by environmental factors such as soil, species life type and growth height. At present, the community succession to coniferous and mixed coniferous forests in the study site is slightly dominated by positively linked species pairs, but the significance level between species pairs is low, the ratio of positive association to negative association is small, the overall association shows insignificant positive linkage, and the *NRI* exhibited a non‐significant aggregation, indicating that the community is still in continuous succession development, and the community has not yet reached the most stable stage. The important value of dominant species in the shrub layer was largely affected by total potassium, pH, and available potassium, and the light (canopy density) also significantly affected *H. heptaphyllum* and *M. malabathricum* . The pH of soil gradually decreased with a change in the slope direction from adret to ubac and increase in the distance from the coastline and island isolation. Slope direction, distance from the coastline, island isolation, temperature, and precipitation indirectly affected the important value of species in shrub layer by directly affecting the pH. Island isolation, temperature, and precipitation also indirectly affected the important value of species in shrub layer by directly influencing soil chemical contents such as N, P, K, and SOM.

## Author Contributions

**Jihong Xiao:** conceptualization (equal), formal analysis (lead), investigation (lead), methodology (equal), validation (equal), visualization (equal), writing – original draft (lead), writing – review and editing (lead). **Qingyan Wen:** conceptualization (equal), data curation (equal), investigation (equal), methodology (equal), project administration (equal), supervision (equal), writing – review and editing (equal). **Zhifei Zhong:** conceptualization (equal), data curation (equal), investigation (equal), methodology (equal), project administration (equal), supervision (equal), writing – review and editing (equal). **Xiting Lin:** conceptualization (equal), data curation (equal), investigation (equal), methodology (equal), project administration (equal), supervision (equal), writing – review and editing (equal). **Yingxue Wang:** conceptualization (equal), data curation (equal), investigation (equal), methodology (equal), project administration (equal), supervision (equal), writing – review and editing (equal). **Yanqiu Xie:** conceptualization (equal), data curation (equal), investigation (equal), methodology (equal), project administration (equal), supervision (equal), writing – review and editing (equal). **Feifan Weng:** conceptualization (equal), data curation (equal), investigation (equal), methodology (equal), project administration (equal), supervision (equal), writing – review and editing (equal). **Qingya Deng:** conceptualization (equal), data curation (equal), investigation (equal), methodology (equal), project administration (equal), supervision (equal), writing – review and editing (equal). **Guochang Ding:** conceptualization (equal), funding acquisition (lead), methodology (equal), project administration (lead), resources (lead), supervision (equal), writing – review and editing (equal). **Chuanyuan Deng:** conceptualization (equal), funding acquisition (lead), methodology (equal), project administration (lead), resources (lead), supervision (equal), writing – review and editing (equal).

## Conflicts of Interest

The authors declare no conflicts of interest.

## Supporting information


Appendices tables

## Data Availability

Data and metadata are archived at the Dryad repository: https://doi.org/10.5061/dryad.w3r2280wv.
