# Supplementary material for: Interspecific Association and Environmental Interpretation of Dominant Species in Shrub Layer of Pinus massoniana Community on Chinese Islands
Source: Ecol Evol. 2024 Dec 5;14(12):e70647. doi: 10.1002/ece3.70647 (PMC11620846; doi:10.1002/ece3.70647)
Supplement: Supplementary file 1 — Appendices tables [file ECE3-14-e70647-s001.zip › ece370647-sup-0003-AppendixS3.docx]

**Key data processing steps**

Redundancy analysis of Figure 2: We used the Canoco 5 software to conduct detrending correspondence analysis (DCA) on the relative importance values of dominant species. Afterword, we used a forward selection method of redundancy analysis (RDA) and Monte Carlo test (499 cycles) were used to screen the environmental factors with significant impact (*P* < 0.05) and study the impact of environmental factors on species distribution.

The niche.width( ), niche.overlap( ) and sp.pair( ) functions in the interspecific linkage analysis package spaa were used to calculate the niche width and niche overlap, respectively. The *χ*2 test, Pearson correlation and Spearman's rank correlation significance tests were performed by the corr. test( ) function in the psych package.
